# Teeth of Children

**Published:** 1876-02

**Authors:** 


### TEETH OF CHILDREN.

No woman can be beautiful whose front teeth are defective or lost: such a blemish to a young girl is an irreparable calamity. A truly wise and loving mother would rather dress her daughter for a whole year in linsey-woolsey, and reduce her diet to the plainest kind—would painfully economize in every direction—rather than let that daughter's teeth be neglected. Many a tooth is lost in early life which would have done service for an age, if the timely care of a judicious dentist had been given it—and this at an expense of only two or three dollars. Cases have come to our knowledge, where teeth have been preserved for more than a quarter of a century, and still appear sound, by skillful plugging

It is a cruelty to neglect the teeth of children. From the time the first teeth begin to be shed, until the tenth year, *every tooth* ought to be most carefully inspected once in three months by a *conscientious* and *skillful* dentist, and thereafter, at least once in six months; for it is known that a decay less than the size of a pin's head will be arrested for a life-time by a well-placed plug; but if delayed a very few months, will be irrecoverably lost.

---

Sugar has become a domestic necessity. It is found in almost all the products of the earth. There is a species of *Palm* which yields sugar-water. It is propagated from the seed; the growth is most flourishing in high lands where there is some frost—but not much; there must be some. One acre will yield from three to five tons of sugar—not requiring any grinding apparatus, a pot and a fire will make it anywhere. A gentleman, whom we know, has sent for several tons of the seed, and we hope for a successful experiment—as sugar is a luxury, which, in some form or other, is essential to our comfort and our health.

